# Outcomes of Living Kidney Donor Candidates and Living Kidney Recipient Candidates with JC Polyomavirus and BK Polyomavirus Viruria

**DOI:** 10.1155/2021/8010144

**Published:** 2021-08-19

**Authors:** Sara Querido, Carolina Ormonde, Teresa Adragão, Inês Costa, Maria Ana Pessanha, Perpétua Gomes, André Weigert

**Affiliations:** ^1^Department of Nephrology, Unit of Renal Transplantation, Hospital de Santa Cruz, Centro Hospitalar de Lisboa Ocidental, Carnaxide, Portugal; ^2^Department of Nephrology, Hospital do Divino Espírito Santo, Ponta Delgada, Portugal; ^3^Department of Clinical Pathology, Laboratory of Clinical Microbiology and Molecular Biology, Centro Hospitalar de Lisboa Ocidental, Lisboa, Portugal; ^4^Centro de Investigação Interdisciplinar Egas Moniz (CiiEM), IUEM, Almada, Portugal

## Abstract

**Introduction:**

Recent data have emerged about a protective association between JCV viruria and chronic kidney disease (CKD). *Material and Methods*. Single-center retrospective cohort study; 230 living kidney donors (LKD) candidates and 59 potential living kidney receptors (LKR) were enrolled. Plasma and urinary JCV and BKV viral loads were measured in all LKD candidates and in nonanuric LKR candidates. Twenty-six living kidney transplant surgeries were performed. LKR were followed in order to evaluate BKV and JCV viremia and urinary viral shedding after KT.

**Results:**

In LKD candidates, JCV viruria was negatively associated with proteinuria of >200 mg/24 hours (JC viruric LKD: 12.5% vs JCV nonviruric LKD: 26.7%, *p*=0.021, OR:0.393; 95% CI: 0.181–0.854). In a multivariate analysis, LKD candidates with JCV viruria had a lower risk of proteinuria of >200 mg/24 hours (*p*=0.009, OR: 0.342, 95% CI: 0.153–0.764), in a model adjusted for age, gender, presence of hypertension, and eGFR <80 mL/min. Prevalence of JCV viruria was higher in LKD candidates when compared with LKR candidates (40.0% vs 1.7%, *p* < 0.001). Among the 26 LKR, 14 (53.8%) KT patients evolved with JCV viruria; 71.4% received a graft from a JCV viruric donor.

**Conclusion:**

Our data corroborate the recent findings of an eventual protective association between JCV viruria and kidney disease, and we extrapolated this concept to a South European population.

## 1. Introduction

Kidney transplantation (KT) is the preferred treatment for eligible patients with end-stage renal disease because it confers substantially longer survival and a better quality of life compared with other renal replacement therapies [1]. Due to organ shortage, living kidney donation becomes an important complement to deceased donor KT. Unilateral nephrectomy inevitably leads to reduced renal mass and function [2], which associates with a rise in blood pressure and increased proteinuria [3, 4]. These factors are responsible for a high risk of cardiovascular and all-cause mortality in general population and, eventually, in living kidney donors (LKD) [5]. Therefore, a careful selection of LKD is crucial, in order to minimize the potential risks associated with living kidney donation [6]. Whereas, many donor baseline characteristics, such as age, predonation estimated glomerular filtration rare (eGFR) and blood pressure, have been included in recent guidelines for the preoperative assessment of LKD [7, 8]; polyomavirus viruria is not routinely measured in either living or cadaveric donors. JC polyomavirus (JCV) and BK polyomavirus (BKV) are human polyomaviruses, which cause asymptomatic childhood infection and persist in various sites including the urinary tract [9] and the central nervous system [10]. Nearly 80% of adults are seropositive for JCV and BKV [11]. Detection of viruria unveils renourinary polyomavirus reactivation [9, 12]; nevertheless, with the exception of immunosuppression, factors that control the balance between latency and reactivation of polyomaviruses are unknown. Previous studies have shown that approximately 30% of adult population shed JCV in urine at any time point [12]. Conversely, patients with chronic kidney disease (CKD) have a significantly reduced urinary shedding rate of JCV, as low as 2.2% [13].

Recent data have emerged about a protective association between JCV viruria and kidney disease. Urinary tract JCV infection was measured in African Americans in order to access relationships with apolipoprotein L1 gene (APOL1)–associated nephropathy. Surprisingly, JCV urinary shedding was associated with lower rates of nephropathy in individuals with APOL1 high-risk genotypes [14]. Additional studies confirmed an association of JCV viruria with protection from CKD in nondiabetic African Americans independently from APOL1 genotype [15], in African Americans with diabetic kidney disease [16] and in Black South Africans with hypertension-attributed CKD [17].

As far as we know, data are scarce about the prevalence and implications of JCV viruria in European, predominantly Caucasian LKD candidates and potential living kidney receptors (LKR) with advanced CKD. We hypothesized that JCV viruria is negatively associated with markers of CKD in donors, such as lower proteinuria and lower estimated glomerular filtration rate (eGFR). We also postulated to find a significantly lower prevalence of JCV viruria in living kidney receptors (LKR) candidates when compared with LKD candidates because its absence have been related with a higher risk of nephropathy.

## 2. Material and Methods

### 2.1. Study Design and Population

In this single-center, retrospective, observational study, we enrolled 230 consecutive LKD candidates and 59 potential LKR, followed in a Kidney Transplant Unit in Portugal.

From November 2013 to August 2020, urine and plasma JCV and BKV viral loads were measured by quantitative, commercial, real-time quantitative polymerase chain reaction (qPCR) in all LKD candidates. Urine and plasma samples were also collected from LKR candidates, except if anuric. In anuric CKD patients, JCV and BKV plasma viral load were not measured either.

In a subgroup of 76 LKD candidates, persistence of polyomavirus viruria was confirmed through a subsequent qPCR measurement, in order to evaluate the consistency of viruria, to avoid false-positive results and to evaluate variations in viral load over time.

As polyomaviruses are transmitted by the oral-fecal route, socioeconomic factors and familial clustering were considered in the analysis of JCV viruria. As LKD candidates are mainly first-degree relatives or spouses of the LKR, we matched the 59 pairs in which both donor and nonanuric receptor had available results for JCV viruria, in order to evaluate possible familial clustering. Conversely, in 171 anuric LKR candidates, it was impossible to evaluate JCV viruria. Prevalence of JCV viruria was compared between matched and nonmatched LKD candidates.

During follow-up period, 26 living kidney transplant surgeries were performed from LKD to nonanuric LKR candidates. LKR were subsequently followed in order to evaluate BKV and JCV viremia and urinary viral shedding after KT. JCV and BKV viremia and viruria wereevaluated every month for the first 6 months and then every 3 months until 2 years after KT.

### 2.2. Data Collection

#### 2.2.1. Living Kidney Donor Candidates

Demographic characteristics of LKD candidates (age, gender, ethnicity) were recorded at the first appointment. Estimated glomerular filtration rate (eGFR) was computed using the Chronic Kidney Disease Epidemiology Collaboration (CKD-EPI) equation [18], and 24-hour proteinuria was also measured at baseline. Blood pressure (BP) was evaluated in all LKD candidates through 24-hour ambulatory blood pressure measurement. Hypertension was defined as mean systolic BP of >130 mg, diastolic BP of >80 mmHg, or a controlled BP under at least one antihypertensive drug.

Outcome data included the association of JCV and BKV viremia or viruria with proteinuria, hypertension, or lower eGFR at baseline.

#### 2.2.2. Kidney Transplant Patients

Demographic characteristics (age, gender), donor/recipient cytomegalovirus (CMV) serologic status, maintenance immunosuppression, length of follow-up, and immunologic risk profile (number of mismatches between donor and recipient, presence of class I and class II anti-HLA antibodies) were also evaluated.

Outcome data included the occurrence of the novo BKV and/or JCV viremia and/or viruria in KT recipients, independent of the donor status.

Tissue typing performed to determine HLA-A, HLA-B, and HLA-DR anti-HLA antibodies was assessed through Luminex assays and using class I and II identification kits.

### 2.3. BKV and JCV Analysis

In both assays, two amplification reactions were performed starting from extracted DNA. For BKV, a specific primer for the region of the Large T antigen gene of BKV and a specific primer for the region of the human beta Globin gene (internal control) were used; for JCV, a specific primer for the Large T antigen region of the JCV gene and a specific primer for an artificial sequence of DNA (internal control) were used. The BKV- and JCV-specific probe with ELITe MGB® technology, labelled with FAM fluorophore, is activated when it hybridizes with the specific product of the BKV and JCV amplification reaction. Viral load is obtained, in each case, through a calibration curve.

### 2.4. Immunosuppressive Regimen

KT recipients received basiliximab or antithymocyte globulin as induction therapy, except if HLA identical, in which situation, no induction therapy was used.

Basiliximab (20 mg IV) was administered in the first and fourth day after KT; antithymocyte globulin (1.25 mg/kg/day IV) was administered since the first day and optimally until the seventh day after KT; methylprednisolone (500 mg on 1st day, 250 mg on 2nd day, 125 mg on 3rd, and 80 mg on 4th day IV after KT) was included in all immunosuppressive induction regimens.

The choice of the immunosuppressive regimen depended, mainly, on patient's immunologic profile (% of panel reactive antibodies; number of HLA mismatches with the donor, preformed donor-specific antibodies).

The maintenance immunosuppressive therapy included, in most patients, tacrolimus, mycophenolate mofetil, and prednisone.

Tacrolimus was administered orally at 0.15 mg/kg/day divided in two doses and adjusted to maintain a target trough concentration between 4 and 10 ng/mL, depending on the time elapsed after KT. Prednisolone was prescribed since the fifth day after KT (0.6 mg/kg) and was tapered to 5 mg/day during the first 3 months after KT. Mycophenolate mofetil (1000 mg orally twice daily) was started after KT and was reduced if adverse events appeared; it was reduced to 1000 to 1500 mg daily dose after the first 3–6 months.

### 2.5. Prophylaxis Regimens

All KT recipients received trimethoprim/sulfamethoxazole (480 mg qd) as *Pneumocystis jirovecii* pneumonia prophylaxis for 1 year.

Valganciclovir (900 mg/d, adjusted to kidney function) was given to patients which induction therapy included antithymocyte globulin and/or rituximab or in receptor CMV IgG-negative/donor CMV IgG-positive pairs for 6 months.

### 2.6. Statistical Analysis

Categorical variables were described as absolute or relative frequencies. Continuous variables were described as mean ± standard deviation (SD), for normally distributed variables, or median and interquartile range (IQR) for nonnormal distributed variables. Proportions were compared using chi-squared test. Differences between clinical data were assessed by Student's *t*-test for unpaired samples for normal variables and Wilcoxon test for continuous data with nonnormal distribution. Binary logistic regression was used for multivariate analysis. A *p* value of <0.05 was considered statistically significant. All statistical tests were performed using the Statistical Package for the Social Sciences (SPSS) 25.0 software (SPSS, Inc, Chicago, IL, USA). Study protocol complies with the Declaration of Helsinki and has obtained full approval from the local clinical research ethics committee.

## 3. Results

Prevalence of JCV and BKV viruria and viremia in living kidney donor candidates and its association with demographic data, blood pressure, eGFR, and proteinuria.

A total of 230 LKD candidates with a mean age of 49 ± 12 years were enrolled in this study. Clinical and demographical data at baseline are detailed in Table 1. The overall prevalence of JCV viruria was 40.0% (*n* = 92), and prevalence of BKV viruria was 4.8% (*n* = 11). BKV and JCV viremia were absent in all LKD candidates. Simultaneous BKV and JCV viruria occurred in 0.9% of LKD candidates (*n* = 2).

JCV viruria was not associated with an eGFR of <80 mL/min (JCV viruric LKD: 19.6% vs JCV nonviruric LKD: 15.9%, *p*=0.480); however, JCV viruria was negatively associated with proteinuria of >200 mg/24 hours (JC viruric LKD: 12.5% vs JCV nonviruric LKD: 26.7%, *p*=0.021, OR: 0.393; 95% CI: 0.181–0.854).

In a multivariate analysis, LKD candidates with JCV viruria had a lower risk of proteinuria of >200 mg/24 hours (*p*=0.009, OR 0.342, 95% CI: 0.153–0.764), in a model adjusted for age, gender, presence of hypertension, and eGFR <80 mL/min (Table 2).

### 3.1. Consistence of JCV Viruria in Different Measures

JCV viruria was reevaluated in 76 (33%) LKD candidates. *De novo* JCV viruria was detected in 3 previously negative LKD candidates, all with a low mean viral load (3.03log_10_). Four individuals who had a first positive JCV with a low viral load (mean 3.08log_10_) became negative in the second evaluation. JCV viruria was consistent in both evaluations in 90.8% of patients (*n* = 69).

Mean JCV urinary viral load was 4.8log_10_ cp/mL in the first measure (*n* = 230) and 4.97log_10_ cp/mL in the second determination (*n* = 76).

### 3.2. Comparison of JCV and BKV Viruria and Viremia between Living Kidney Donor Candidates and Living Kidney Recipient Candidates

JCV and BKV viruria and viremia were measured in 59 LKR candidates who had a urine output higher than 500 mL/day. Mean eGFR was 19.1 ± 6.4 mL/min. Only 2 patients had polyoma viruria (one JCV and one BKV). Both of them had CKD stage 5 but still not under renal replacement therapy.

Prevalence of JCV viruria was significantly higher in LKD candidates when compared with LKR candidates (40.0% vs 1.7%, *p* < 0.001). No differences were observed between BKV viruria prevalence in LKD and LKR candidates (4.8% vs 1.7%, *p*=0.470). JCV and BKV viremia was absent in all LKR candidates.

### 3.3. Assessment of Possible Familial Clustering for JCV Viruria

Fifty-nine LKD candidates were matched with their LKR candidates in the evaluation of JCV viruria. The prevalence of JCV viruria in matched LKD candidates was 45.8% (*n* = 27). No difference was found in the prevalence of JCV viruria when compared with matched and nonmatched LKD candidates (45.8% vs 38.0%, *p*=0.355).

### 3.4. Evaluation of JCV and BKV Viruria and Viremia after Kidney Transplantation

Demographical and clinical data of the 26 living kidney transplanted patients are described in Table 3. Two kidney transplant recipients did not receive induction immunosuppression because donors were HLA identical.

During the follow-up period, 5 (19.2%) patients developed BKV viruria, 4 of them in the first 2 months after KT. Furthermore, 3 of them subsequently developed BKV viremia. Pretransplantation BKV viruria was absent in all their respective 5 matched donors. Fourteen (53.8%) KT patients evolved with JCV viruria, 78.6% (*n* = 11) within 2 months posttransplant. One LKD patient had previous JCV viruria. Ten *de novo* JCV viruric patients (71.4%) received a graft from a JCV viruric donor. Two LKR patients developed transient low-grade JCV viremia. JCV and BKV coinfection was observed in 2 (7.7%) patients. Because BKV and JCV infection was detected in a small number of KT patients, no additional statistical analysis was performed. Thus, the role of immunosuppression therapy and donor/recipient immunological profile remains unclear.

## 4. Discussion

The seroprevalence of BKV and JCV infections in adults ranges from 60 to 100% worldwide [12, 19]. Both JCV and BKV appear to establish benign latent infection in renourinary epithelium with periodic reactivation and viral excretion in urine [9, 12]. Nevertheless, asymptomatic urinary shedding of BKV and JCV has shown conflicting results. In a recent report from Kuwait [20], 165 potential kidney donors were tested for the presence of BKV and JCV viruria and viremia. Despite that 42% of individuals tested positive for polyomavirus DNA, due to low viral loads in most specimens, the authors found a prevalence of only 13% for JCV viruria and 1% of BKV viremia by qPCR. In a Brazilian study [13], the prevalence of JCV viruria in a healthy control group was 20.1%, whereas BKV prevalence was 2.2%. Meanwhile, Rodrigues et al. [21] noted a 23.9% urinary excretion of JCV and a 1.8% of BKV virus in the general population in Portugal. However, in a group of 120 African American nonnephropatic individuals [15], prevalence of JCV and BKV viruria varied, respectively, between 40% and 0% for APOL1 renal risk genotype group vs 48.8% and 1.2% for APOL1 nonrisk genotype group. The wide variability in polyomavirus prevalence in different studies is due to low viral load present in healthy individuals and the lack of sensitive assays, as qPCR, in older studies. Our results are in line with previous studies, considering the higher incidence of JCV viruria (40%) compared with BKV viruria (4.8%). Similar to previous studies [20], BKV and JCV viremia were absent in all studied samples. Thus, despite that human polyomavirus are common infections, it seems that individuals with normal immune systems are capable to suppress viremia.

Coinfection with more than one polyomavirus is a rare phenomenon. In a study in KT patients, infection with one polyomavirus was inversely associated with infection by the other virus, possibly through direct inhibition or competition for the same molecular pathways involved in DNA replication [22]. Similarly, coinfection rate was rare in our LKD candidates (0.87%).

Contrary to the majority of studies, in which only an occasional sample is available, we aimed to confirm the persistence of virus shedding and variations in viral load over time. In a subgroup of 76 LKD candidates, virus-specific urinary loads were measured in two different times. JCV viruria was consistent between the 2 measures in 90.8% of patients, and mean viral load was identical. JCV DNA remained stable over time; therefore, an isolated positive sample was enough to define the state of JCV urinary carrier.

In our study, the prevalence of JCV viruria was significantly higher in LKD candidates when compared with LKR candidates (40.0% vs 1.7%, *p* < 0.001). As far as we known, this is the first report that demonstrates an inverse association between JCV urinary shedding and CKD in a South European population. The current findings are strengthened by some recent reports where a potential protective association between JCV viruria and kidney disease was underlined. Divers at al. [14] tested whether infection by JCV and BKV modulated the association between APOL1 and the development of nephropathy. They collected samples from 300 first-degree relatives of African Americans with nondiabetic end-stage renal disease. After adjusting for age, sex, and African ancestry proportion, authors found that the presence of JCV viruria in patients with increased risk of APOL1-associated nephropathy (2 APOL1 risk variants) was negatively associated with albuminuria and CKD (eGFR <60 mL/min/1.73 m^2^). BKV viruria was not associated with kidney disease. Concerning these results, authors postulated that JCV may interact with APOL1 genotypes to modulate kidney disease risk.

The potential protective effect of JCV urinary shedding against the development of CKD in African American individuals, independently of APOL1 genotype, was validated in a subsequent analysis [15]; both mild and more severe CKD patients were included to prevent possible confounding effects of reduced nephron mass or urinary excretion of detectable viral shedding. As JCV was present in 45.8% of nonnephropathy controls and 8.75% of CKD cases regardless of APOL1 renal risk genotype status, authors postulated a robust CKD protective effect by JCV (OR: 0.15; 95% CI: 0.06–0.42).

These results were further extended to African Americans with diabetic kidney disease (DKD) [16]. This report, which enrolled 335 individuals (148 patients with DKD vs 187 controls), corroborated that JCV viruria was inversely associated with DKD (OR: 0.56, 95% CI: 0.35–0.91; *p*=0.02).

Also, Nqebelele et al. [17] reproduced the same findings in Black South Africans with hypertension-attributed CKD. Beyond a group of CKD patients and a nonrelated control group, the study included a control group of first-degree relatives of CKD cases. Despite an identical socioeconomic status score between CKD patients and their first-degree relatives, prevalence of JCV viruria was significantly different between these groups (nonrelated controls 36%; first-degree relatives 20%, and CKD patients 3%, *p* < 0.001). The absence of JCV viruria was a strong predictor of CKD (OR 43.43 95% CI 7.39–255.2, *p* > 0.001).

Our results are in line with previous reports. We compared the prevalence of JC viruria in LKD candidates who had a paired LKR candidate, with LKD candidates who were nonmatched due to the absence of urinary samples of their respective LKR candidates. JCV viruria prevalence was similar between both groups of LKD candidates. Thus, as LKD candidates are mainly first-degree relatives or spouses, the possibility that the absence of JCV viruria in the LKR candidates was related to socioeconomic factors or family clustering was excluded.

We also report a prevalence of JCV and BKV viruria and viremia in healthy individuals vs CKD patients similar to previous studies. The prevalence of JCV viruria was significantly higher in LKD candidates when compared with LKR candidates (40% vs 1.7%, *p* < 0.001), and no differences were observed between BKV viruria prevalence between both groups. A Brazilian study [13] reported significantly lower frequencies of JC viruria in Brazilians with end-stage renal disease relative to nonnephropathic controls (3.9% vs 20.1%, *p* < 0.0001). The authors considered that two factors might be involved in this finding: first, high urinary urea concentrations could inhibit the amplification of segments of the polyomavirus sequence; second, a reduced volume and density of the urine and a reduced renal mass of end-stage renal disease patients could be associated with decreased cell scaling in the urine, consequently leading to reduced viral load in the samples [13].

However, in most recent studies [14, 15], the protective association between JCV viruria with kidney disease is valid even in patients with less severe renal impairment. In line with these studies, our 59 CKD patients with preserved diuresis had a mean eGFR of 19 ± 6 mL/min and only 2 of them had polyoma viruria.

As in other cross-sectional observational studies, it is difficult to establish a cause-effect relationship for the potential beneficial role of JCV in the protection against CKD. So, it is impossible to determine whether JCV viruria has a direct effect on antagonizing deleterious pathways in CKD or whether it is an epiphenomenon that reflects a common complex mechanism responsible for both kidney injury and JCV replication blockade.

Specific pathways potentially contributing to JCV-reduced urinary shedding remain to be characterized. According to Freedman et al. [15], different potential mechanisms need to be considered. First, CKD could lead to a reduced production of molecules crucial to promote JCV urinary reactivation, or inversely, CKD could stimulate the production of inhibitory factors that might block the expression of JCV in the urinary tract. A mechanism of renal inflammation leading to renal tubular atrophy, parenchymal sclerosis, and inhibition of JCV replication seems to be plausible. Second, reduced JCV expression could be a marker of an immune and inflammatory response, which leads simultaneously to progressive kidney injury and suppression of JCV viruria. Third, enhanced JCV viruria could translate an altered host immune response leading to reduced inflammation and an inability to clear the virus from the renourinary tract. Fourth and less probably, JCV reactivation in non-CKD patients could competitively inhibit other nephrotoxic viruses.

We were not able to demonstrate an association between eGFR and JCV viruria in LKD candidates, probably because the reduced number of patients with eGFR of <80 mL/min. Nevertheless, JCV viruria was negatively associated with 24-hour proteinuria of >200 mg and that association persists when possible confounding factors such as gender, age, presence of hypertension, and eGFR of <80 mL/min were included in multivariate analysis. Divers et al. [14] had already demonstrated an association between albumin to creatinine ratio over 30 mg/g and JCV urinary shedding. Longitudinal prospective studies are required to better understand if the absence of JCV viruria could be a potential marker for further development of CKD in uninephrectomized LKD.

Twenty-six KT patients were followed after living KT. We found a prevalence of BKV viruria of 19.2% and a prevalence of JCV viruria of 53.8%. In the large majority of cases, polyomavirus viruria appeared as early as 2 months after KT, which is in line with previous reports. In our series, JCV was the most frequently detected polyomavirus. Nevertheless, available data on KT patients remain conflicting. Even though some authors have shown that JCV viruria could be as high as 56.8% and greater than BKV [23], the majority of reports suggest that BKV viruria is more frequent than JCV viruria [22, 24]. As observed in the group of nonimmunosuppressed patients, coinfection with both polyomavirus was a rare phenomenon, empowering the theory of a possible inhibitory interaction between BKV and JCV in KT patients [22].

During the follow-up period, 5 (19.2%) patients developed BKV viruria; however, BKV viruria was absent in all their 5 matched donors, suggesting reactivation. Conversely, 71.4% of *de novo* JCV viruric KT patients received a graft from a JCV viruric donor. Literature regarding sources of polyomavirus in renal allograft recipients are scarce and conflicting [23, 25, 26]. Notwithstanding, the tendency is to accept that polyomavirus urinary viral shedding may be transmitted by the allograft. We could only extrapolate this transmission for JCV. However, it was impossible to know if JCV urinary shedding in KT patients was due to their own reactivation of a latent virus that has already been able to express after the recovery of kidney function or if it was due to a direct donor/recipient transmission. Pretransplantation donor and recipient BKV IgG levels could have been helpful. Nevertheless, it seems that a preserved kidney function is crucial to express JCV in urine. Considering the reduced number of KT performed, it was impossible to achieve any definitive consideration. Also, giving the short follow-up period, the impact of polyomavirus urinary shedding in kidney function and development of polyomavirus-associated nephropathy was not studied. The role of immunosuppression therapy and donor/recipient immunological profile in JCV and BKV reactivation remains unclear.

## 5. Conclusions

As far as we know, this was the first cohort study where JCV and BKV viruria and viremia were evaluated in matched LKD and LKR candidates, complemented by follow-up of JCV and BKV reactivation in KT receptors. Our data corroborates the recent findings of an eventual protective association between JCV viruria and kidney disease, and we extended this concept to a South European, predominantly Caucasian population. Due to the low incidence of JCV viruria in LKR candidates, more sophisticated statistical analysis, including multivariate analyses, was impossible to perform. Prospective longitudinal studies are needed in order to understand the role of JCV in health and disease.

## Figures and Tables

**Table 1:** Clinical and demographical data of viruric and notviruric living kidney donor candidates

Characteristics	All LKD (*n* = 230)		LKD with JC viruria (*n* = 92)		LKD without JC viruria (*n* = 138)		*p* value
Age–mean ± SD, years	49.1	±12.0	50.3	±12.2	47.9	±11.8	0.083
Male gender, *n* (%)	78	(33.9)	36	(39.1)	42	(30.4)	0.201
Caucasian, *n* (%)	206	(89.6)	85	(92.4)	121	(87.7)	0.280
Hypertension-*n* (%)	50	(21.7)	24	(26.1)	26	(18.8)	0.197
Serum creatinine, median (IQR), mg/dL	0.8	(0.7–0.9)	0.8	(0.7–0.9)	0.8	(0.7–0.9)	0.360
eGFR <80 mL/min/1.73 m^2^, *n* (%)	40	(17.4)	18	(19.6)	22	(15.9)	0.480
24-hour proteinuria >200 mg, *n* (%)	42	(21)	10	(12.5)	32	(26.7)	**0.021**

LKD: living kidney donor candidates; SD: standard deviation; eGFR: estimated glomerular filtration rate. For 24-hour proteinuria >200 mg the number of LKD with available results are 200 (missing data in 30 LKD candidates).

**Table 2:** JCV viruria-LKD candidates relationships (logistic regression).

	Standard error	*p*-value	OR	95% CI	
				Inferior	Superior
Age	0.015	0.485	1.010	0.982	1.040
Gender	0.317	0.059	1.820	0.977	3.389
Hypertension	0.385	0.245	1.564	0.735	3.328
eGFR <80 mL/min	0.440	0.859	1.081	0.457	2.560
24-hour proteinuria >200 mg	0.410	0.009	0.342	0.153	0.764

Dependent variable: JCV viruria; CI: confidence interval; OR: odds ratio; eGFR: estimated Glomerular Filtration Rate.

**Table 3:** Demographical and clinical data of living kidney transplanted patients.

Living kidney recipients	*N* = 26
Male (*n*/%)	17/65.4
Age (mean; SD) (years)	45 ± 12.4 (23–69)
Length of follow-up (mean; SD) (months)	36.5 ± 21.5 (9–79)

Induction IMS (*n*/%)	
** **No induction	2/7.7
** **Bas + TAC + MMF + Pred	14/53.8
** **TIMO + TAC + MMF + Pred	10/38.5

Maintenance IMS (*n*/%)	
** **TAC + MMF + Pred	19/73
** **EVE + MMF + Pred	7/27
** **Donor CMV IgG+ (*n*/%)	20/76.9
** **Recipient CMV IgG+ (*n*/%)	19/73
** **Valganciclovir prophylaxis (*n*/%)	17/63

HLA mismatches with the donor (mean ± SD)	
** **A or B	2.15 ± 2.1
** **DR	1.2 ± 0.7

Anti-HLA antibodies (*n*/%)	
** **Class I	10/38.5
** **Cass II	13/50
** **Class I + II	9/34.6

SD: standard deviation; IMS: immunosuppression; Bas: basiliximab; TAC: tacrolimus; MMF: mycophenolate mofetil; Pred: prednisone: TIMO: thymoglobulin; EVE: everolimus; CMV: cytomegalovirus; IgG: immunoglobulin G; HLA: human leukocyte antigen.

## Data Availability

The data used to support the findings of this study are available from the corresponding author upon request.

## References

[1] Kasiske B. L., Snyder J., Matas A., Collins A. (2000). The impact of transplantation on survival with kidney failure. *Clinical Transplants*.

[2] Ibrahim H. N., Foley R., Tan L. (2009). Long-term consequences of kidney donation. *New England Journal of Medicine*.

[3] Boudville N., Ramesh Prasad G. V., Knoll G. (2006). Meta-analysis: risk for hypertension in living kidney donors. *Annals of Internal Medicine*.

[4] Garg A. X., Muirhead N., Muirhead N. (2006). Proteinuria and reduced kidney function in living kidney donors: a systematic review, meta-analysis, and meta-regression. *Kidney International*.

[5] Mjøen G., Hallan S., Hartmann A. (2014). Long-term risks for kidney donors. *Kidney International*.

[6] Ahmadi A. R., Lafranca J. A., Claessens L. A. (2015). Shifting paradigms in eligibility criteria for live kidney donation: a systematic review. *Kidney International*.

[7] European Renal Best Practice Transplantation Guideline Development Group, Abramowicz D., Cochat P. (2013). Guideline. *Nephrology Dialysis Transplantation*.

[8] Lentine K. L., Kasiske B. L., Levey A. S. (2017). KDIGO clinical practice guideline on the evaluation and care of living kidney donors. *Transplantation*.

[9] Randhawa P., Brennan D. C. (2006). BK virus infection in transplant recipients: an overview and update. *American Journal of Transplantation*.

[10] Elsner C., Dörries K. (1992). Evidence of human polyomavirus BK and JC infection in normal brain tissue. *Virology*.

[11] Andrews C. A., Shah K. V., Daniel R. W., Hirsch M. S., Rubin R. H. (1988). A serological investigation of BK virus and JC virus infections in recipients of renal allografts. *Journal of Infectious Diseases*.

[12] Egli A., Infanti L., Dumoulin A. (2009). Prevalence of polyomavirus BK and JC infection and replication in 400 healthy blood donors. *The Journal of Infectious Diseases*.

[13] Pires E. P., Bernardino-Vallinoto C. V., Alves D. M. (2011). Prevalence of infection by JC and BK polyomaviruses in kidney transplant recipients and patients with chronic renal disease. *Transplant Infectious Disease*.

[14] Divers J., Núñez M., High K. P. (2013). JC polyoma virus interacts with APOL1 in African Americans with nondiabetic nephropathy. *Kidney International*.

[15] Freedman B. I., Kistler A. L., Skewes-Cox P. (2018). JC polyoma viruria associates with protection from chronic kidney disease independently from apolipoprotein L1 genotype in African Americans. *Nephrology Dialysis Transplantation*.

[16] Kruzel-Davila E., Divers J., Russell G. B. (2019). JC viruria is associated with reduced risk of diabetic kidney disease. *Journal of Clinical Endocrinology & Metabolism*.

[17] Nqebelele N. U., Dickens C., Dix-Peek T., Duarte R., Naicker S. (2019). JC virus and APOL1 risk alleles in Black South Africans with hypertension-attributed CKD. *Kidney International Reports*.

[18] Levey A. S., Stevens L. A., Schmid C. H. (2009). A new equation to estimate glomerular filtration rate. *Annals of Internal Medicine*.

[19] Reploeg M. D., Storch G. A., Clifford D. B. (2001). Bk virus: a clinical review. *Clinical Infectious Diseases*.

[20] Chehadeh W., Kurien S. S., Nampoory M. R. (2013). Molecular characterization of BK and JC viruses circulating among potential kidney donors in Kuwait. *BioMed Research International*.

[21] Rodrigues C., Pinto D., Medeiros R. (2007). Molecular epidemiology characterization of the urinary excretion of polyomavirus in healthy individuals from Portugal-a Southern European population. *Journal of Medical Virology*.

[22] Cheng X. S., Bohl D. L., Storch G. A. (2011). Inhibitory interactions between BK and JC virus among kidney transplant recipients. *Journal of the American Society of Nephrology*.

[23] Signorini L., Dolci M., Favi E. (2020). Viral genomic characterization and replication pattern of human polyomaviruses in kidney transplant recipients. *Viruses*.

[24] Saundh B. K., Baker R., Harris M., Hale A. (2016). A prospective study of renal transplant recipients reveals an absence of primary JC polyomavirus infections. *Journal of Clinical Virology*.

[25] Urbano P. R. P., Nali L. H. d. S., Oliveira R. d. R. (2019). Variable sources of Bk virus in renal allograft recipients. *Journal of Medical Virology*.

[26] Grellier J., Hirsch H. H., Mengelle C. (2018). Impact of donor BK polyomavirus replication on recipient infections in living donor transplantation. *Transplant Infectious Disease*.

